# PtdIns (3,4,5) P3 Recruitment of Myo10 Is Essential for Axon Development

**DOI:** 10.1371/journal.pone.0036988

**Published:** 2012-05-10

**Authors:** Huali Yu, Nannan Wang, Xingda Ju, Yan Yang, Dong Sun, Mingming Lai, Lei Cui, Muhammad Abid Sheikh, Jianhua Zhang, Xingzhi Wang, Xiaojuan Zhu

**Affiliations:** Key Laboratory of Molecular Epigenetics of Ministry of Education, Institute of Cytology and Genetics, Northeast Normal University, Changchun, China; MRC, University College of London, United Kingdom

## Abstract

Myosin X (Myo10) with pleckstrin homology (PH) domains is a motor protein acting in filopodium initiation and extension. However, its potential role has not been fully understood, especially in neuronal development. In the present study the preferential accumulation of Myo10 in axon tips has been revealed in primary culture of hippocampal neurons with the aid of immunofluorescence from anti-Myo10 antibody in combination with anti-Tuj1 antibody as specific marker. Knocking down Myo10 gene transcription impaired outgrowth of axon with loss of Tau-1-positive phenotype. Interestingly, inhibition of actin polymerization by cytochalasin D rescued the defect of axon outgrowth. Furthermore, ectopic expression of Myo10 with enhanced green fluorescence protein (EGFP) labeled Myo10 mutants induced multiple axon-like neurites in a motor-independent way. Mechanism studies demonstrated that the recruitment of Myo10 through its PH domain to phosphatidylinositol (3,4,5)-trisphosphate (PtdIns (3,4,5) P3) was essential for axon formation. In addition, in vivo studies confirmed that Myo10 was required for neuronal morphological transition during radial neuronal migration in the developmental neocortex.

## Introduction

Typical mature neurons have a highly polarized structure with a long axon to transmit information and multiple short dendrites to receive information. The formation of polarized neurons is the first step for the establishment of neuronal circuits [1]. In the classical primary culture system, without obvious external polarity cues, hippocampal neurons extend active lamellipodia and filopidia (stage 1), and these dynamic outgrowths then develop into several relatively symmetric minor processes (stage 2). Within the first 24 h after plating, one neurite driven by a dynamic reorganization of the cytoskeleton elongates rapidly into a characteristic axon (stage 3), while the other neurites become dendrites [2]. Selective localizations of molecules determine axon-dendrite differentiation by persistently supplying the elongating axon with growth promoting proteins [3], which is triggered by activation of phosphoinositide 3-kinase (PI3K) and the accumulation of its lipid product of PtdIns (3,4,5) P3 at the tip of future axon [4], [5], [6], [7]. Importantly, PtdIns (3,4,5) P3, a membrane lipid, is sufficient to stimulate actin cytoskeleton remodeling in coordination with neuronal polarity and axon elongation [8], [9], [10], [11]. A recent study showed that accumulation of actin in the outgrowing axon was increased in the growth cone as well as in the whole axon shaft [12]. Despite the significant progress in identification of numerous actin binding proteins to regulate axon development [13], [14], [15], however, the mechanism of axon formation is still not fully understood.

Class X myosin (myosin X, Myo10), a molecular motor, localizes at the tip of filopodia and other actin-rich peripheral protrusions and is critical for filopodium formation and cell motility [16]. It contains an N-terminal motor domain that binds to actin filaments and hydrolyzes ATP for its movement along the actin filament [17]. In the neck domain, three IQ motifs bind calmodulin and calmodulin-like proteins [18]. The C-terminal region contains the following domains: three pleckstrin homology (PH) domains binding phosphatidylinositol (3,4,5)-trisphosphate (PtdIns (3,4,5) P3) [19], a MyTH4 domain for binding microtubules [20] ], and a FERM domain serving to transport proteins toward the tip of filopodia. These cargo proteins including Mena/VASP [21], β-integrin [22], DCC [23], ALK6 [24], and VE-Cadherin [25] enable Myo10 to function in filopodium extension and adhesion. Recent studies showed that the localization of Myo10 at the tip of filopodia was regulated by PtdIns (3,4,5) P3 and PtdIns (3,4,5) P3 binding was required for Myo10 movement on actin filaments [26]. It is roughly known that silencing of Myo10 in vivo by microRNA impaired axon outgrowth in chick commissural neurons in our earlier study [23]. However, deciphering the cellular and molecular mechanism underlying the effects of Myo10 for axon development remains a valid question.

In this study, we investigated the distribution and function of Myo10 in cultured hippocampal neurons. Interestingly, reduced outgrowth of axon with the loss of Tau-1-positive phenotype was observed in Myo10 knockdown neurons. Importantly, cytochalasin D (Cyto. D) rescued the axon defect caused by reduction of Myo10 expression. Gain-of-function studies indicated that Myo10 induced multiple axon-like neurites in a motor-independent manner. The axogenic effects were regulated by PtdIns (3,4,5) P3 and its binding with Myo10 through PH recruitment was essential for axon development. Finally, studies in vivo revealed that Myo10 was required for neuron morphological transition from multipolar to bipolar.

## Results

### Myo10 is accumulated in the tip of developing axon

**Figure 1:**
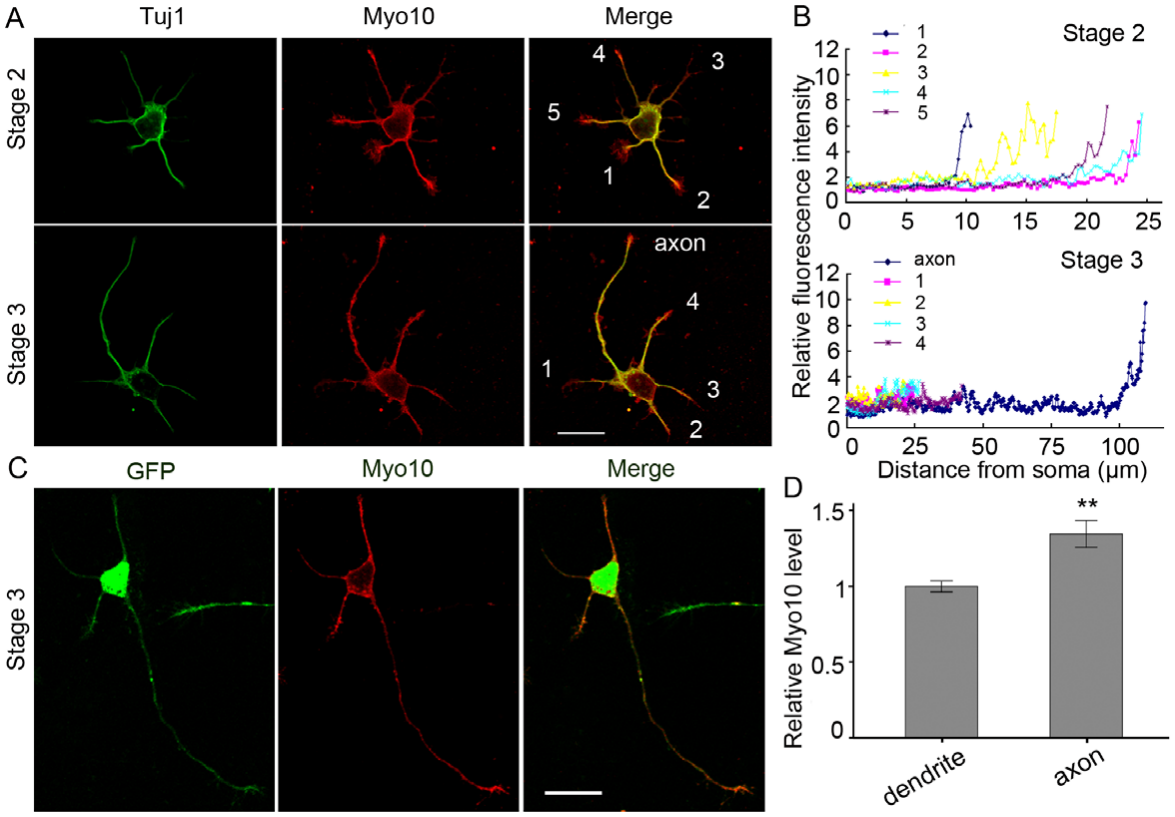
Distribution of Myo10 in cultured hippocampal neurons. A, Hippocampal neurons at 24 h after plating were immuno-stained with antibodies against Myo10 and Tuj1. B, Quantitative analysis of Myo10 distribution in a representative stage 2 and a stage 3 neuron. C, Neurons were transfected with pEGFP-C1 to visualize the entire neurite and stained with Myo10 antibody at stage 3 after culture for 24h. D, Relative immune-fluorescence intensity of Myo10 versus GFP in axon tips. For quantification, average value of Myo10/GFP in dendrites was normalized to 1±0.04. Scale bar, 20 µm. **P<0.01.

To explore the role of Myo10 in neuronal development, the immunofluorescence of double labeling in cultured hippocampal neurons was performed 24 h after plating with anti-Myo10 antibody as well as anti-Tuj1 antibody, the specific beta-tubulin marker. In stage 2 neurons, Myo10 was distributed uniformly in the neurites and accumulated in the tips of most processes. By stage 3, Myo10 seemed to be more abundant in the tips of longest neurites which were destined to the nascent axons (Fig. 1A and B). Furthermore, neurons were transfected with pEGFP-C1 as a fluorescent marker to visualize the neurites [27]. The ratio of Myo10 versus GFP (relative intensity) in dendrites at stage 3 neurons was normalized as 1.0±0.04, whereas that in axons was 1.35±0.09, which showed that Myo10 staining was more abundant in the nascent axons than in other neurites (Fig. 1C and D, n = 50, *P*<0.01). The preferential accumulation of Myo10 in the developing axon tips provided a clue that Myo10 was involved in the axon outgrowth.

**Figure 2:**
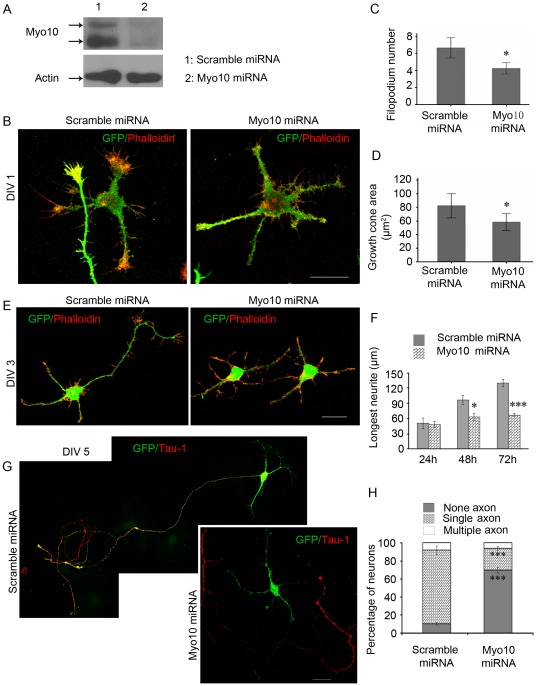
Loss of Myo10 function inhibits the formation of axon. A, Westen blot showing the down-regulation of Myo10 by Myo10 miRNA in cultured rat hippocampal neurons. B, Neurons transfected with scramble miRNA or Myo10 miRNA were stained with TRITIC-Pholloidin at DIV 1. C and D, Quantitative analysis of filopodium number and area of growth cone. E, Myo10 miRNA decreases the length of axon-like neurites at DIV 3. F, Quantitative analysis of the average length of the longest neurites 24, 48 and 72 h after plating. G, The neurons were stained with anti-Tau-1 antibody to identify axon at DIV 5. H, Percentages of neurons with none, single and multiple axons. Scale bar, 20 µm. *P<0.05; ***P<0.001.

**Figure 3:**
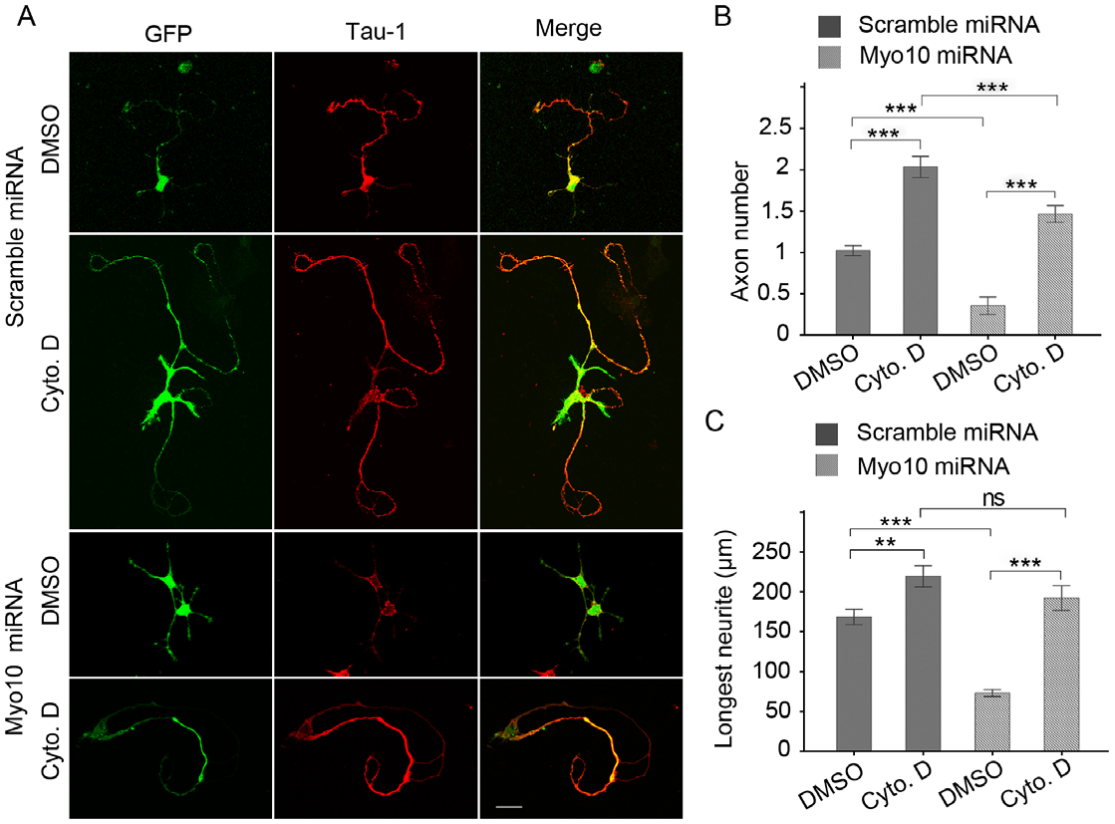
Application of Cyto. D rescues the axon defect caused by Myo10 knockdown. A, Neurons transfected with scramble miRNA and Myo10 miRNA respectively were cultured in the presence of DMSO or 1 µm Cyto. D for 8–72 h culture and labeled with anti-Tau-1 antibody at DIV 5. B and C, Quantitative analysis of average number of axons and the length of the longest neurites in the presence of Cyto. D. Scale bar, 20 µm. **P<0.01; ***P<0.001; ns, no significant difference.

**Figure 4:**
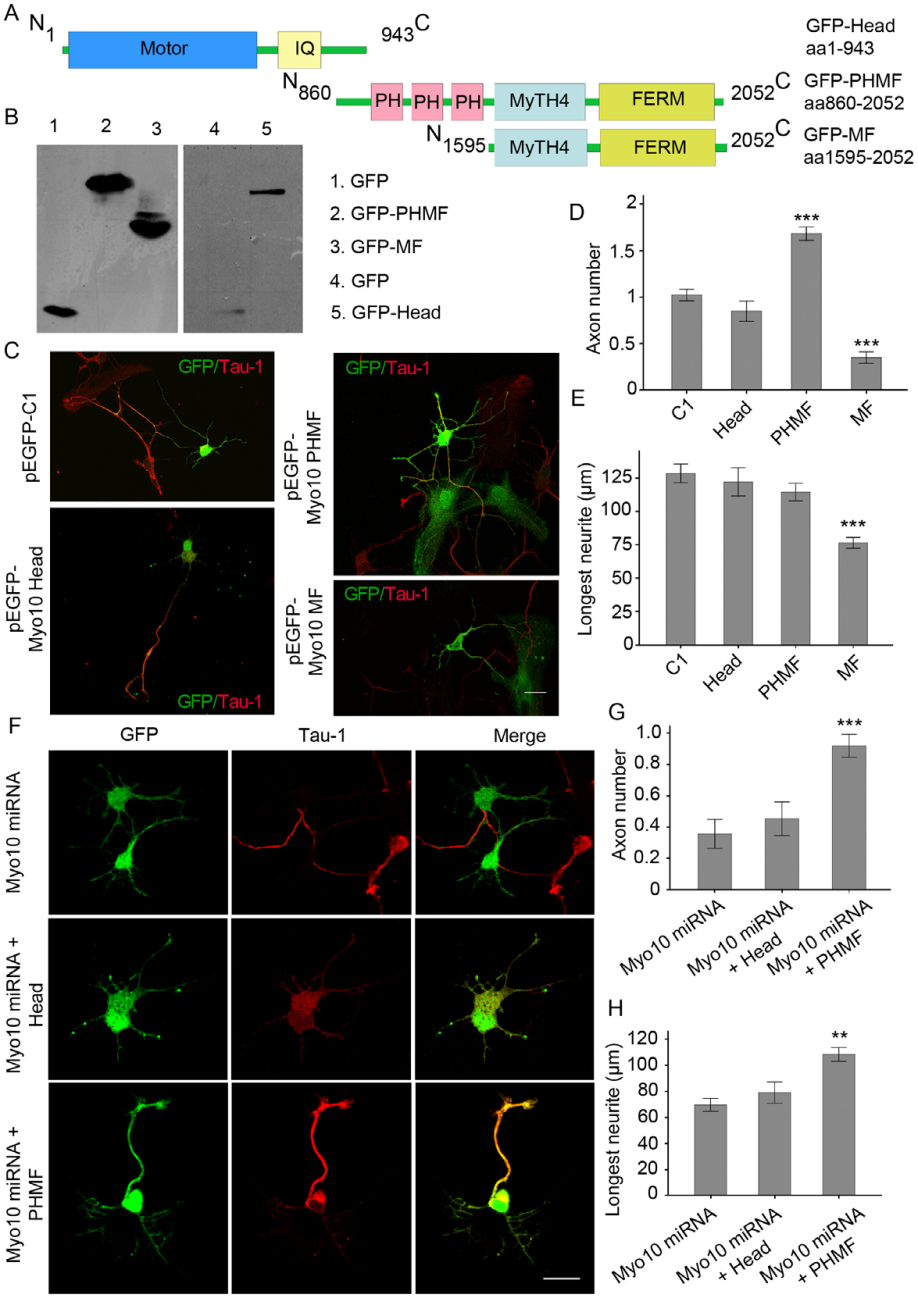
Overexpression of Myo10 PHMF induces the formation of multiple axon-like neurites. A, Schematic representations of Myo10 Head, Myo10 PHMF and Myo10 MF. B, Western blot detects expression of GFP, GFP-Head, GFP-PHMF and GFP-MF. C, Neurons transfected with pEGFP-C1, pEGFP-Myo10 Head, pEGFP-Myo10 PHMF and pEGFP-Myo10 MF respectively were labeled with anti-Tau-1 antibody at DIV 3. D and E, Quantitative analysis of average number of axons and the longest neurites. F, Myo10 PHMF is sufficient for axon formation. Neurons transfected with Myo10 miRNA together with Myo10 Head and Myo10 PHMF respectively were stained with Tau-1 antibody at DIV 3. G and H, Quantitative analysis of average number of axons and average length of the longest neurites. Scale bar, 20 µm. **P<0.01; ***P<0.001.

**Figure 5:**
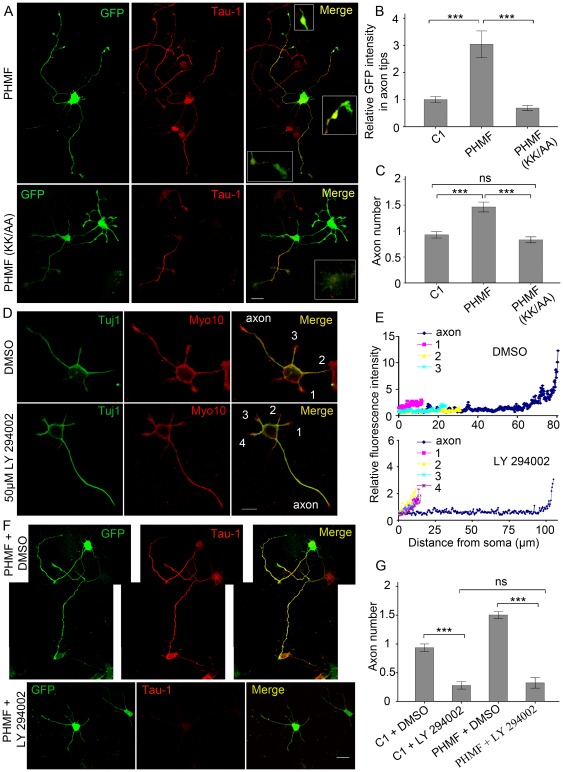
Myo10 is regulated by PtdIns (3,4,5) P3 in axon development. A, Neurons transfected with Myo10 PHMF and Myo10 PHMF (1225KK/6AA) were stained with anti-Tau-1 antibody at DIV 3. B, Quantitative analysis of the average GFP intensity in the entire growth cones of axons. The average value of GFP intensity in pEGFP-C1 transfected neurons was normalized to 1±0.1. C, Quantitative analysis of average number of axon-like neurites. D, Hippocampal neurons short-term treated with DMSO and 50 µM LY 294002 were immuno-stained with antibodies against Myo10 and Tuj1 at 24 h after plating. E, Quantitative analysis of Myo10 distribution in representative neurons. F, Neurons transfected with Myo10 PHMF were cultured in DMSO and 50 µM LY 294002. G, Quantitative analysis of average number of axon in the presence of DMSO and LY 294002. Scale bar, 20 µm. ***P<0.001; ns, no significant difference.

**Figure 6:**
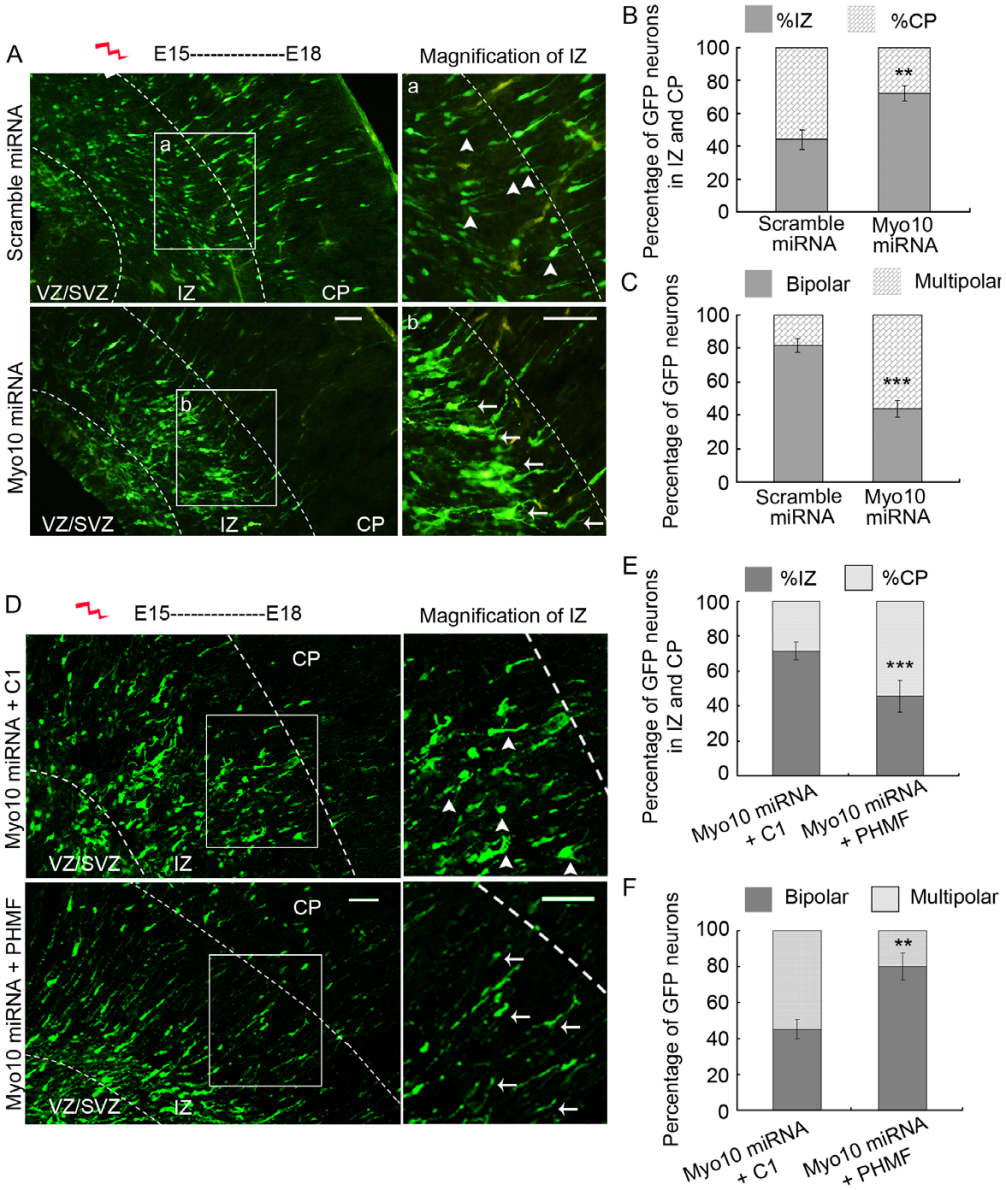
Myo10 miRNA inhibits neuronal transition from multipolar to bipolar in the IZ. A, Images of sections of embryonic neocortex transfected with scramble miRNA and Myo10 miRNA, respectively. Higher magnification of the boxed regions is shown in upper IZ. B, Percentage of GFP-neurons in the IZ and CP. C, Percentage of GFP-neurons with bipolar or multipolar morphologies in the IZ. D, Images of sections of embryonic neocortex transfected with Myo10 miRNA together with pEGFP-C1 or PHMF, respectively. Higher magnification of the boxed regions is shown in upper IZ. E, Percentage of GFP-neurons in the IZ and CP. F, Percentage of GFP-neurons with bipolar or multipolar morphologies in the IZ. Scale bar, 50 µm. **P<0.01; ***P<0.001.

### Knockdown of Myo10 impairs axon development

To clarify the role of Myo10 during neurite development, loss-of-function experiments were performed by RNA interference (RNAi). A Myo10 miRNA expression vector that specifically targeted rat Myo10 was described in our previously work [23]. Rat hippocampal neurons were transfected with scramble miRNA and Myo10 miRNA immediately after dissociation. The GFP signal was detectable after about 6–10 hours culture. The silence effect for endogenous Myo10 protein was confirmed by Western blot at day in vitro (DIV) 3 and a great reduction of protein level was observed in transfected neurons relative to the cells which transfected with scramble miRNA (Fig. 2A). Having proved the effectiveness of Myo10 miRNA construct, dissociated hippocampal neurons were transfected immediately, cultured and analyzed at DIV 1, 3 and 5, respectively. At DIV 1, the actin-rich growth cones were less developed by reducing Myo10, with the number of filopodia as well as the area of growth cone significantly decreased (Fig. 2B, C and D, n = 50, *P*<0.05). At DIV 3, the majority of neurons transfected with Myo10 miRNA failed to extend a long axon-like structure (Fig. 2E). The growth for the longest neurite was hampered for 48 h culture (Fig. 2F, scramble miRNA: 96.84±9.14 µm; Myo10 miRNA: 63.07±6.87 µm, n = 50, *P*<0.05) and the impairment was more significant for 72 h culture (Fig. 2F, scramble miRNA: 120.89±7.72 µm; Myo10 miRNA: 67.05±2.92 µm, n = 100, *P*<0.001) indicating that the impairment of neurite outgrowth took place between 24–48 h culture, the time point for axon rapid elongation. The total number of primary neurites showed no significant difference (Fig. S1A, scramble miRNA: 5.53±0.17; Myo10 miRNA: 5.10±0.16, n = 100, *P*>0.05). Then, at DIV 5, with axon marker anti-Tau-1 antibody, we analyzed the percentage of neurons with none, single and multiple axons to assess neuronal polarization. The results showed that the majority of the neurons expressing Myo10 miRNA lost the characteristic neuronal morphology. There were only 24.1%±2.0% neurons displaying single Tau-1-positive axon in Myo10 miRNA transfected neurons, much lower compared to 81.4%±4.9% in scramble miRNA transfected neurons (Fig. 2G and H, Fig. S1B, none axon: 10.3%±1.5% in scramble miRNA group; 69.9%±3.1% in Myo10 miRNA group, n = 200, *P*<0.001). In usually, there were a small fraction of neurons with multiple axons in vitro culture [27]. Also, we observed 6.0%±3.6% multipolar neurons in Myo10 miRNA groups and 8.2%±4.3% in scramble miRNA group, with no significant decrease after Myo10 knockdown (Fig. 2H, *P*>0.05). Taken together, these results suggested that Myo10 was required for axon development.

### Cytochalasin D rescues the axon defect

It has been postulated that sustained growth of future axon is triggered through a labile actin cytoskeleton. Hence, we asked whether the effect of Myo10 knockdown on axon development could be eliminated by application of actin-depolymerizing drug Cyto. D. A single dose of Cyto. D (1 µM) was added to culture medium 8 h after plating and the neurons were incubated in the presence of the drug to DIV 3. The results showed that in the presence of Cyto. D, neurons typically displayed multiple axon-like neurites, which was consistent with previous studies, verifying the effectiveness of Cyto. D to induce axon formation (Fig. 3A and B, scramble miRNA + DMSO: 1.02±0.40; scramble miRNA + Cyto. D: 2.04±0.76, n = 100, *P*<0.001). Notably, both number of Tau-1 positive axon and the longest neurite were restored significantly for Cyto. D incubation in the Myo10 knockdown neurons (Fig. 3, axon number: 0.43±0.69 for Myo10 miRNA + DMSO neurons, and 1.47±0.77 for Myo10 miRNA + Cyto. D neurons; longest neurite: 73.17±4.46 µm for Myo10 miRNA + DMSO and 192.46±15.39 µm for Myo10 miRNA + Cyto. D, n = 150, *P*<0.001). Moreover, the loss of Myo10 weakened the role of Cyto. D in supernumerary axons induction (Fig. 3, axon number: 2.04±0.76 for scramble miRNA + Cyto. D neurons; 1.47±0.77 for Myo10 miRNA + Cyto. D, *P*<0.001), indicating that Myo10 was a potential regulator of cytoskeleton for axon formation.

### Ectopic Myo10 expression induces multiple axon-like neurites in a motor-independent way

To determine the minimal region of Myo10 sufficient for axon development, a series of enhanced GFP (EGFP)-Myo10 truncated mutations were constructed and tested by Western blots (Fig. 4A, B). Then, neurons were transfected with the labeled constructs and analyzed at DIV 3. Statistical results showed that neurons transfected with pEGFP-Myo10 Head extended a single long axon-like neurite. However, pEGFP-Myo10 PHMF induced multiple Tau-1 positive neurites with the number of axons 1.68±0.072. The pEGFP-Myo10 MF transfected neurons lost typical Tau-1 positive neurites and the longest neurites was decreased to 76.43±4.04 µm (Fig. 4C, D and E, n = 250, *P*<0.001). These results suggested that PHMF domain was specifically required for axon formation, whereas Myo10 MF domain might competitively inhibit the role of endogenous Myo10. To further test the function of Head domain and confirm the role of PHMF, rescue experiment was performed. Dramatically, overexpression of Myo10 PHMF reversed the axon defect caused by Myo10 miRNA with both the number of axons and the longest neurite increased significantly (Fig. 4F–H and Fig. S2, n = 250, *P*<0.01). These morphologic changes observed gave us two clues: First, Myo10-induced axon formation was motor domain independent, which suggested a new mechanism different from the traditional view of Myo10 in cytoskeleton regulation. Second, the PH domains of Myo10 were involved in the underlying mechanism.

### PtdIns (3,4,5) P3 is a regulator of Myo10 in axon development

To dissect the molecular mechanism, the existence of an upstream regulator that interacted with the PH domain of Myo10 was proposed. The data from cell lines reveals that Myo10 can be recruited through its PH domain to PtdIns (3,4,5) P3, which is generated by PI3K activation. As PtdIns (3,4,5) P3 plays a critical role in axon formation, we wondered whether the function of Myo10 in axon formation was linked to PtdIns (3,4,5) P3 recruitment. Mutagenesis of the conserved residues in the second PH domain (KK1225/6AA), a key binding site with PtdIns (3,4,5) P3, was performed in Myo10 PHMF (Fig. S3A) according to previous study [26]. For quantitative analysis of the GFP intensity in the entire growth cone of axon, the average value of GFP intensity in pEGFP-C1 transfected neurons was normalized to 1±0.1. In PHMF transfected neurons, the relative GFP intensity was 3.04±0.49 and the number of axon accordingly increased to 1.60±0.064. However, neurons transfected with Myo10 PHMF (KK1225/6AA) were unable to induce supernumerary axons and as expected, PHMF (KK1225/6AA) was less targeted to axon tips (Fig. 5A, B and C, n = 200, *P*<0.001). Thus, PtdIns (3,4,5) P3 binding is necessary for Myo10 function in axon development.

We next inhibited the production and accumulation of PtdIns (3,4,5) P3 with PI3K inhibitor LY 294002. 20 µM and 50 µM LY 294002 were respectively added to culture medium 16 h after plating and the neurons transfected with pEGFP-C1 were incubated in the presence of the drug to 24 h. The accumulation of Myo10 in axon tips was not significantly reduced at the dose of 20 µM LY 294002. Prominent decrease of Myo10 in axon tips was detected in the case of 50 µM LY 294002 incubation (Fig. S3B and C). The distribution of Myo10 in nascent axons in two representative neurons showed that inhibition of PtdIns (3,4,5) P3 synthesis with PI3K inhibitor disrupted the preferential accumulation of Myo10 in nascent axon (Fig. 5D and E). Furthermore, in the presence of 50 µM LY 294002 from 8 h to DIV 3, the majority of neurons transfected with pEGFP-C1 lost Tau-1 positive axons and the longest neurites phenotype (Fig. S3D and E), suggesting that less accumulation of PtdIns (3,4,5) P3 led to similar effect with Myo10 knockdown. It was confirmed that with PI3K inhibitor Myo10 PHMF was not capable of inducing more Tau-1 positive axon and the length of longest neurite was decreased (from 118.15±6.70 µm to 66.64±7.76 µm, Fig. S3D and E, n = 150, *P*<0.001). In conclusion, disruption of Myo10 binding with PtdIns (3,4,5) P3, either by mutation of Myo10 or by inhibition of PtdIns (3,4,5) P3 accumulation, impaired the capacity of Myo10 to promote axon characteristic, showing the recruitment of Myo10 to PtdIns (3,4,5) P3 is required for axon development.

### Myo10 miRNA interrupts neurons transition from multipolar to bipolar in vivo

To confirm the role of Myo10 in axon development, we introduced Myo10 miRNA into mouse embryos in embryonic day 15 (E15) via in utero electroporation. Thereafter, the distribution of GFP-positive neurons was analyzed at E18. More than half of scramble miRNA transfected neurons (55.8±6%) migrated into the cortical plate (CP). In contrast, most of Myo10 miRNA transfected neurons (71.7±4.4%) remained in the intermediate zone (IZ) (Fig. 6A and B, n = 5 independent brains for each condition, *P*<0.01). The morphological changes of neurons were analyzed by comparing the percentage of transfected neurons in unipolar/bipolar and multipolar in the IZ. As shown in Figure 6, about 81.5±4.1% of control neurons had reestablished a bipolar morphology. However, only about 44.1±5.1% of Myo10 miRNA transfected neurons exhibited a bipolar morphology, and 55.9±5.1% of neurons still arrested in a multipolar morphology in upper IZ (arrow) (Fig. 6C, n = 5 independent brains for each assay, *P*<0.001), suggesting that Myo10 was specifically involved in regulating bipolar morphological transition during radial neuronal migration. Importantly, the defect of neuronal migration was rescued by coelectroporation of Myo10 miRNA and PHMF with 54.3%±9.1% neurons migrating into CP relative to 28.6%±5.0% neurons into CP in Myo10 miRNA and pEGFP-C1 co-transfected neurons (Fig. 6D and E, n = 5, *P*<0.001). As expected, the morphological transition from multipolar to bipolar was restored in co-expression of Myo10 miRNA with PHMF neurons (Fig. 6D and F, bipolar neurons: 45.1%±5.4% for Myo10 miRNA + C1 group, 80.0%±7.5% for Myo10 miRNA + PHMF group, n = 5, *P*<0.01). Taken together, the above results confirmed that Myo10 was involved in neuronal morphogenesis in vivo.

## Discussion

Myo10 is known as a molecular motor, which has been shown to promote filopodia extension under the modulation of PtdIns (3,4,5) P3 in COS 7 kidney carcinoma cells [26]. It has also been previously shown that Myo10 is capable of supporting cortical neurite initiation in the absence of Ena/VASP [28]. In this report we provide direct experimental evidences that Myo10 motor-independent neurite outgrowth is critical for axon formation.

In neurons with reduction of Myo10 expression by RNA interference, the outgrowth of axons is severely hampered. The growth cone of the axon exhibits reduction of filopodium number and growth cone area. Actin-depolymerizing Cyto. D reverts the Myo10 knockdown phenotype, resulting in neurons extending one or more long axons. The restore of axon outgrowth in Myo10 knockdown neurons with Cyto. D suggested that Myo10 was not necessary for microtubule assembly in axon outgrowth. At the growth cone periphery, extension of actin-based structures, filopodia and lamellipodia, led to the growth cone expansion. Then, microtubules engorged from central zone into the former transitional region. One possible explanation for Cyto. D rescue effect is that Myo10 promotes actin dynamics in the growth cone. Loss of Myo10 impaired filopodia extension and growth cone expansion. Thus, close actin arcs prevented microtubule protruding to peripheral region of the growth cone. Cyto. D caused local destabilization of actin for microtubule extension.

Interestingly, overexpression of the PH-MyTH4-FERM domains (PHMF) without the Myo10 motor induced the extension of supernumerary axons with expression of Tau-1 phenotype. It is revealed that Myo10 can promote axons outgrowth in a motor-independent manner. In the nervous system, there is a headless isoform of Myo10, which lacks almost the whole motor domain [29]. Since overexpression of headless Myo10 abolished filopodium-promoting activity and inhibited filopodium extension [16], it was generally considered that headless Myo10 functioned as a dominant negative protein to spatially limit the activities of full-length Myo10. However, the present data provide a proof that headless Myo10 can initiate and expedite axon formation in cultured hippocampal neurons complementing the observations of developmental regulations of headless Myo10 in brain development as revealed in situ hybridization [29]. There might be dual roles of Myo10 relying on distinct binding modes as Myo10 Head prefers localizing to the tip of filopodia and Myo10 PHMF to the whole growth cone (Fig. 4).

The existence of PH domains endows Myo10 with properties to bind PtdIns (3,4,5) P3. PI3K activation generates accumulations of PtdIns (3,4,5) P3 in the tips of developing axons, which is an essential step in promoting axon development [4], [5], [7], [30]. Myo10 can be recruited by PtdIns (3,4,5) P3 via the second PH domain and accumulates in the axons, especially the tips of axon, a similarly distribution phenotype with PtdIns (3,4,5) P3 [4] and phosphatidylinositol transfer proteins (PITPs) [7]. Besides, we find that the role of Myo10 in axon formation relies on PI3K activation. Thus, our data support the hypothesis that Myo10 is recruited by accumulated PtdIns (3,4,5) P3 to the tips of nascent axons, where PtdIns (3,4,5) P3 serves to regulate actin polymerization for axon polarity and extension. This is in agreement with the previous reports that binding of PtdIns (3,4,5) P3 to the PH domain activates the mechanical and cargo transporter activity of Myo10 [31] and is necessary for Myo10 promotion of filopodia [26].

How PtdIns (3,4,5) P3 recruitment of Myo10 is coupled to actin polymerization for outgrowth? Given the cargo-binding role of Myo10 MyTH4-FERM domain, it may target the certain cargo molecular near to the membrane for local actin reorganazation as nucleation of actin filaments occurs just beneath the plasma membrane. The potential candidate cargos may include Mena/VASP which promotes polymerization by antagonize capping proteins. Alternatively, a local positive feedback loop of PI3K-Cdc42-Par3/Par6/aPKC-Rac1-PI3K emerges as a driving force for axon development [1], [32]. Myo10 might be implicated in this cascade. For example, Myo10 might also be implicated in axon development by associating with protein kinase C-ε, a component of the Par complex [33]. Overexpression of the Myo10 PHMF domain might enhance the positive feedback loop at tips of supernumerary neurites by modulating the actin cytoskeleton.

In utero electroporation provides a paradigm for gene manipulation under physiological conditions. Postmitotic neurons in the neocortex are generated near the ventricle, first display a multipolar morphology in the lower part of IZ, subsequently transform into a polarized bipolar morphology in the upper part of IZ and start locomotion migration along the radial glial fiber and finally locate in CP. The transition from multipolar to bipolar in IZ is critical for radial neuronal migration as the leading process of bipolar neurons leads the way towards CP by attaching to the fibers of radial glia cells. Studies in vivo revealed the involvement of Myo10 in neuronal transition from multipolar to bipolar and loss of Myo10 resulting in less neurons radially migrating into CP. As we all know, morphological development depends on cytoskeleton coordination. Considering that Myo10 is required for axon outgrowth in vitro, it is conceivable that Myo10 drives axon-dendrite shaping by regulating cytoskeleton reorganization in vivo. Further in vivo studies are required to clarify the effect of Myo10 on neuronal development in detail.

## Materials and Methods

### Reagents

Anti-Myo10 antibody was prepared as previously described [23]. Mouse monoclonal antibodies against Tuj1, Tau-1 and HA were purchased from Abcam (Cambridge), Sigma Aldrich (Saint. Louis) and Millipore (Temecula), respectively. Rabbit polyclonal antibody against GFP was purchased from Santa Cruz Biotechnology (California). Alexa Fluor 488- or 546-coupled secondary antibodies against mouse or rabbit IgG and HRP-conjugated secondary antibodies against mouse or rabbit IgG were from Invitrogen (Hong Kong). Phalloidin conjugated with TRITIC, Cyto. D and LY 294002 were purchased from Sigma Aldrich (Saint. Louis).

### Constructs

The cDNAs of Myo10 mutants were subcloned into mammalian expression vectors pEGFP-C1 fused with EGFP at the amino-terminus. All constructs were confirmed by sequencing. Point mutation of PH domain in Myo10 PHMF (KK1225/6AA) was generated with quikChange lightning site-directed mutagenesis kit (Stratagen). And PHMF (KK1225/6AA) was simply termed PHMF (KK/AA). Myo10 miRNA was generated by the BLOCK-iT Lentiviral miRNA Expression System (Invitrogen, Carlsbad) as described in our previous work [23], and EGFP was expressed with the miRNA from the same plasmid implying that all GFP-cells expressed miRNA.

### Neuronal culture and transfection

Dissociated culture of hippocampal neurons were prepared as described previously [23]. In brief, hippocampi were dissected from P0 rats, digested with 0.125% trypsin at 37°C for 25 min and dissociated by pipetting in DMEM/F12 with 10% fetal bovine serum. Immediately after dissociation, neurons were transfected by electroporation using the Rat Neuron Nucleofector Kit (Amaxa). For electroporation, 2–2.5×10^6^ neurons were resuspended in 100 µl of Nucleofectamine solution containing 3 µg of plasmid and plated at 1.5×10^5^ per cover slip. After neurons attached to the substrate (about 6–8 h), the medium was changed to Neurobasal medium with 2% B27 supplement and 2 mM glutamine.

### Immunofluorescence

Neurons were fixed in 4% paraformaldehyde for 15 min at room temperature, permeabilized with 0.1% Triton X-100 for 10 min and blocked in 2% bovine serum albumin for 1 h in 0.01 M phosphate-buffered saline (PBS; pH 7.4). Subsequently, cells were incubated with primary antibodies diluted in the blocking solution for 2 h and washed three times with PBS. And they were incubated with appropriate fluorochrome-conjugated secondary antibodies for 1 h and washed 3 times.

### Western blot analysis

Total proteins were extracted in RIPA lysis buffer supplemented with protease inhibitor cocktail. Protein samples were separated by SDS-PAGE, then transferred to polyvinylidene difluoride (PVDF). The membranes were incubated with the primary antibodies overnight at 4°C, then washed with PBST and incubated with secondary antibodies for 1 h at room temperature. Detection was facilitated by electrogenerated chemiluminescence (ECL, Amersham) solution and exposure to X-Ray films. To test the silence effect of Myo10 miRNA, Western blot was performed with cultured rat hippocampal neurons.

### In utero electroporation

All mice were handled according to the Guidelines for the Care and Use of Laboratory Animals. The project was approved by the Institution Animal Care and Use Committee of Northeast Normal University. Plasmids were microinjected into the lateral cerebral ventricle of E15 mouse embryos through the uterine wall. Then, 30 V square-wave pulse was delivered across the head for 5 times and interval 50 msec by electrodes in parallel with the median raphes. Embryos were then allowed to develop to E18. The transfected brains were fixed with 4% PFA/PBS overnight at 4°C. The brain sections were sectioned with a freezing microtome at about 12 µm.

### Image analysis and quantification

Images were captured using Olympus FV1000 Viewer confocal microscope (Tokyo, Japan). Morphometric analysis of neuronal shape parameters including filopodium number, growth cone area, number of neurites and axonal lengths were performed using the ImageJ software. The growth cone was defined as the distal part of the neurite where the diameter is twice that of the neurite itself [34]. The filopodium was defined as actin-protrusion from the growth cone periphery with a specified threshold of at least 0.5 µm [35]. The area of largest growth cone per neurons was analyzed and the filopodium number in the largest growth cone was determined. Fluorescence intensity was measured using with Image-Pro Plus software. The ratio of Myo10 versus GFP in a square of 5×5 pixels within each process tip was determined from fluorescence intensities of both channels after background subtraction [36]. Statistical comparison of the distribution of Myo10 in neurites and in axon tips were performed using ANOVA test, which was also used to analyze filopodium number, growth cone area, the length of the longest neurites, the average number of axon and the accumulation of GFP. χ2 test was performed for comparision of percentage of neurons with none, single and multiple axons between scramble miRNA and Myo10 miRNA transfected neurons. The percentage of GFP-neurons in IZ or CP and percentage of bipolar or multipolar neurons were compared between scramble miRNA and Myo10 transfected neocortex following χ2 test. The data were presented as means ± s.e.m.

## Supporting Information

Figure S1 A, Quantitative analysis of neurites number. B, Neurons transfected with scramble miRNA and Myo10 miRNA respectively were stained with anti-Tau-1 antibody at DIV 5. Single colour images (GFP and Tau-1) for assessment of the distribution of Tau-1 in Myo10-depleted neurons. Scale bar, 20 µm. ns, no significant difference.
(TIF)

Figure S2 A, HA-tagged PHMF and Myo10 miRNA were co-electroporated into neurons with the total amount of 6 µg at mole ratio of 1∶1 for 2–2.5×10^6^ neurons. At DIV 2, the neurons were stained with GFP and HA antibodies to show expression of microRNA sequence and PHMF respectively. B, Percentage of neurons with single GFP, single HA and GFP/HA staining. GFP and HA co-expressed neurons accounted for more than 80% of all transfected neurons and neurons expressing HA alone or GFP alone was only about 10.0% and 6.4%, respectively, which was not enough to interfere with the statistical results. Scale bar, 20 µm. ***P<0.01; ns, no significant difference.
(TIF)

Figure S3 A, Myo10 sequence (NM_019472). The KK in red colour were mutated to AA in green. B, Hippocampal neurons transfected with pEGFP-C1 to visualize the entire neurite were immuno-stained with Myo10 antibody at 24 h after plating with short-term application of DMSO, 20 µM LY 294002 and 50 µM LY 294002. C, Relative immune-fluorescence intensity of Myo10 in axon tips. The average value of Myo10/GFP in axon tips in the presence of DMSO was normalized to 1±0.18. D, Neurons transfected with pEGFP-C1 were cultured in DMSO and 50 µM LY 294002. E, Quantitative analysis of average length of the longest neurites. Scale bar, 20 µm. **P<0.01; ***P<0.001; ns, no significant difference.
(TIF)
