# Supplementary figures and images for: PtdIns (3,4,5) P3 Recruitment of Myo10 Is Essential for Axon Development
Source: PLoS One. 2012 May 10;7(5):e36988. doi: 10.1371/journal.pone.0036988 (PMC3349655; doi:10.1371/journal.pone.0036988)

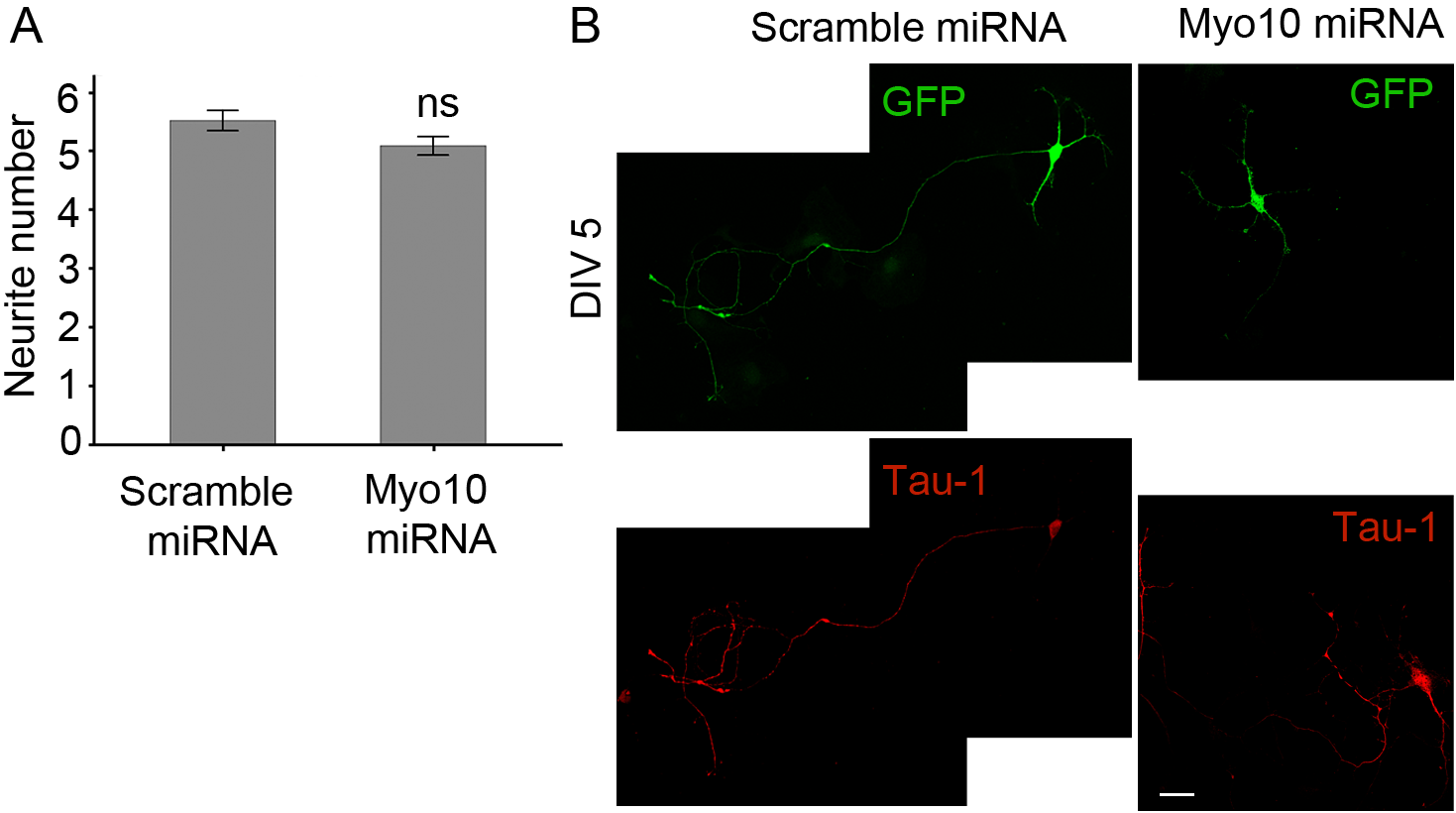

Supplement: Figure S1 — A, Quantitative analysis of neurites number. B, Neurons transfected with scramble miRNA and Myo10 miRNA respectively were stained with anti-Tau-1 antibody at DIV 5. Single colour images (GFP and Tau-1) for assessment of the distribution of Tau-1 in Myo10-depleted neurons. Scale bar, 20 µm. ns, no significant difference. (TIF) [file pone.0036988.s001.tif]

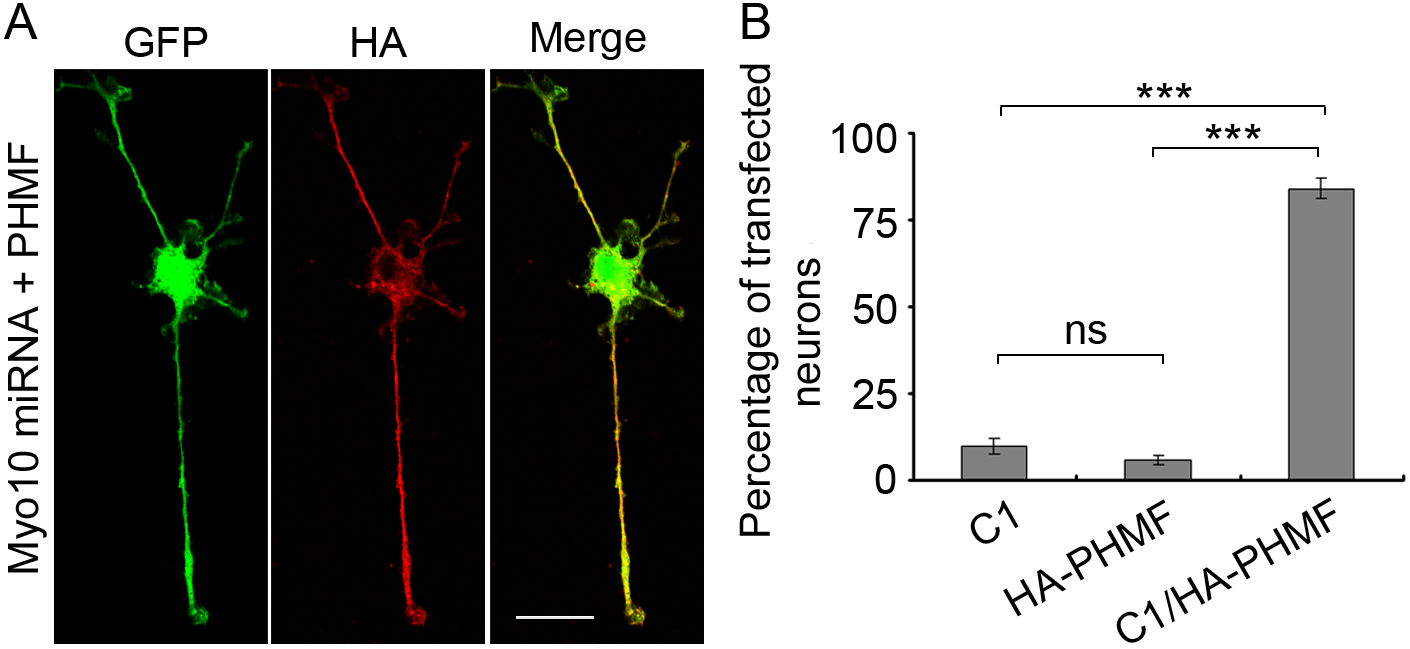

Supplement: Figure S2 — A, HA-tagged PHMF and Myo10 miRNA were co-electroporated into neurons with the total amount of 6 µg at mole ratio of 1∶1 for 2–2.5×10^6^ neurons. At DIV 2, the neurons were stained with GFP and HA antibodies to show expression of microRNA sequence and PHMF respectively. B, Percentage of neurons with single GFP, single HA and GFP/HA staining. GFP and HA co-expressed neurons accounted for more than 80% of all transfected neurons and neurons expressing HA alone or GFP alone was only about 10.0% and 6.4%, respectively, which was not enough to interfere with the statistical results. Scale bar, 20 µm. ***P<0.01; ns, no significant difference. (TIF) [file pone.0036988.s002.tif]

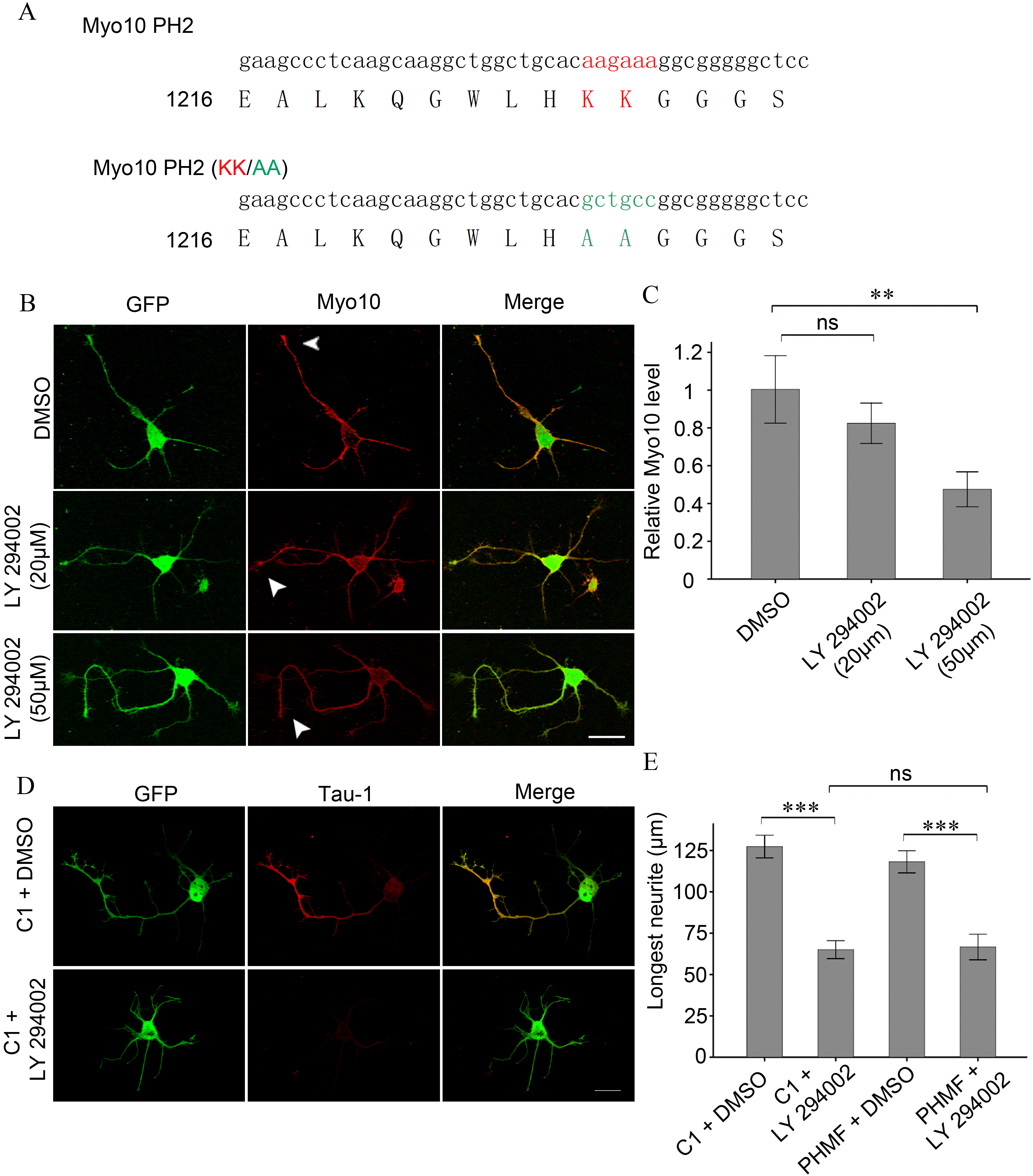

Supplement: Figure S3 — A, Myo10 sequence (NM_019472). The KK in red colour were mutated to AA in green. B, Hippocampal neurons transfected with pEGFP-C1 to visualize the entire neurite were immuno-stained with Myo10 antibody at 24 h after plating with short-term application of DMSO, 20 µM LY 294002 and 50 µM LY 294002. C, Relative immune-fluorescence intensity of Myo10 in axon tips. The average value of Myo10/GFP in axon tips in the presence of DMSO was normalized to 1±0.18. D, Neurons transfected with pEGFP-C1 were cultured in DMSO and 50 µM LY 294002. E, Quantitative analysis of average length of the longest neurites. Scale bar, 20 µm. **P<0.01; ***P<0.001; ns, no significant difference. (TIF) [file pone.0036988.s003.tif]
